# Incidence of Healthcare-Associated Infections in a Neonatal Intensive Care Unit before and during the COVID-19 Pandemic: A Four-Year Retrospective Cohort Study

**DOI:** 10.3390/jcm12072621

**Published:** 2023-03-30

**Authors:** Mariateresa Ceparano, Antonio Sciurti, Claudia Isonne, Valentina Baccolini, Giuseppe Migliara, Carolina Marzuillo, Fabio Natale, Gianluca Terrin, Paolo Villari, The Collaborating Group

**Affiliations:** 1 Department of Public Health and Infectious Diseases, Sapienza University of Rome, 00185 Rome, Italy; 2 Department of Maternal and Child Health, Policlinico Umberto I, Sapienza University of Rome, 00161 Rome, Italy

**Keywords:** neonatal intensive care unit (NICU), healthcare associated infection (HAI), COVID-19

## Abstract

The COVID-19 pandemic may have had an impact on healthcare-associated infection (HAI) rates. In this study, we analyzed the occurrence of HAIs in a neonatal intensive care unit (NICU) of the Umberto I teaching hospital in Rome before and during the pandemic. All infants admitted from 1 March 2018 to 28 February 2022 were included and were divided into four groups according to their admission date: two groups before the pandemic (periods I and II) and two during the pandemic (periods III and IV). The association between risk factors and time-to-first event was analyzed using a multivariable Cox regression model. Over the four-year period, a total of 503 infants were included, and 36 infections were recorded. After adjusting for mechanical ventilation, birth weight, sex, type of delivery, respiratory distress syndrome, and previous use of netilmicin and fluconazole, the multivariable analysis confirmed that being hospitalized during the pandemic periods (III and IV) was the main risk factor for HAI acquisition. Furthermore, a change in the etiology of these infections was observed across the study periods. Together, these findings suggest that patient management during the pandemic was suboptimal and that HAI surveillance protocols should be implemented in the NICU setting promptly.

## 1. Introduction

Healthcare-associated infections (HAIs) are among the most serious preventable complications in neonatal intensive care units (NICUs) [1]. Preterm infants are susceptible to HAIs because of their immature immune systems and prolonged need for indwelling catheters [2]; the risk of these diseases is inversely associated with birth weight and gestational age and increases with time spent in care [3]. The most common type of HAI in NICUs is bloodstream infection (BSI), which can occur in isolation or in association with urinary tract infections and meningitis [3]. The main microorganisms responsible for HAIs include *Staphylococcus aureus*, coagulase-negative *Staphylococci*, and *Enterococci*. In addition, recent years have recorded a considerable increase in HAIs sustained by Gram-negative bacteria and fungi, especially *Candida* spp., which are mainly responsible for ventilator-associated pneumonia (VAP) and urinary tract infections, but also for peritonitis and meningitis [3,4].

According to a study conducted in 2016/2017 [5] using the European Centre for Disease Prevention and Control protocol, the prevalence of HAIs in Italian NICUs was around 5%. However, the organizational challenges experienced during the COVID-19 pandemic may have limited the effectiveness of traditional HAI prevention and control efforts, resulting in an increase in their incidence, as already reported for the adult intensive care unit (ICU) of Umberto I teaching hospital of Rome [6] or in a recent systematic review that investigated *Pseudomonas aeruginosa* bacteremia [7]. Indeed, despite NICUs having one of the lowest COVID-related caseloads among all ICUs, they are still vulnerable to indirect adverse effects of the COVID-19 pandemic [8]. During the pandemic, NICUs faced challenges that were different in nature from those in pediatric and adult ICUs; there were particular concerns relating to clinical workflows and parent–child interactions [9,10], including uncertainty in how to address the risk of exposure for mothers and their babies, reorganization of processes and operations aimed at minimizing risks to staff and patients, and frequent changes in clinical scenarios [8].

While the impact of the pandemic on incidence rates of nosocomial infections in adult ICUs has been investigated [6,11,12,13], data from the NICU setting are scarce. A few indirect effects of the pandemic on NICUs have been described, such as psychological distress or obstacles in implementing family-centered care [14]. In addition, a reduction in hospital-wide availability of alcohol-based hand rubs was reported to be associated with an increase in the rate of central line-associated BSIs in a single-center study [8]. However, few studies have investigated the impact of pandemic-related measures on the incidence of HAIs in preterm infants admitted to NICUs [8,15]. Therefore, the aim of this study was to analyze the occurrence of HAIs in neonates admitted to the NICU of Umberto I teaching hospital of Rome before and during the COVID-19 pandemic and to identify key factors associated with HAI onset.

## 2. Materials and Methods

### 2.1. Setting

In this cohort study, we retrospectively analyzed patients hospitalized in the NICU of Umberto I teaching hospital of Rome from 1 March 2018 to 28 February 2022. Patients were followed until discharge or 23 March 2022. The NICU has a total of six beds in which healthcare providers take care of critically ill babies born in this hospital or coming from other hospitals in Rome and the Lazio region via the Neonatal Emergency Transport Service. We followed the STROBE guidelines to report our findings. Microorganisms’ antimicrobial susceptibility profiles were defined according to the classification proposed by Magiorakos et al. [16] (if applicable), whereas coagulase-negative *Staphylococci* were considered as susceptible or resistant to oxacillin and/or glycopeptides [17]. The institutional ethics board of the Umberto I teaching hospital of Rome approved this study (protocol no. 888/2022).

### 2.2. Data Collection

Data about patients hospitalized in the NICU were retrieved from the prospective patient-based HAI surveillance system that has been conducted in the unit since March 2014 by the Department of Public Health and Infectious Diseases of Sapienza University of Rome. The surveillance personnel routinely review and collect data from patients’ medical records, including clinical data and microbiological findings, on a weekly basis using a standardized form. All neonates hospitalized in the NICU for at least 48 h are included and followed until their discharge from the NICU. Data on date of birth, date of admission and discharge, sex, gestational age, birth weight (BW), type of delivery, admission diagnosis (preterm birth, twin pregnancy, or respiratory distress syndrome), exposure to invasive devices (days of central line catheterization, including umbilical catheter, central venous catheter and peripherally inserted catheter, and days of mechanical ventilation), use of antimicrobial agents (days), site of infection, date of infection onset, and microorganism isolated are routinely collected. An infection is considered to be healthcare-associated if it occurs 48 h after birth or admission. The surveillance system records central/umbilical line-associated bloodstream infections (CLABSIs) and ventilator-associated pneumonia (VAP) and any other type of infection that occurs during hospitalization, the diagnosis of which is determined by an infectious disease specialist. All infections are defined according to the standard diagnostic criteria published by the Center for Disease Control and Prevention (CDC), adapted to neonatal pathology [18].

### 2.3. Statistical Analysis

Patients were divided into four groups according to their admission date: period I, from 1 March 2018 to 28 February 2019; period II, from 1 March 2019 to 29 February 2020; period III, from 1 March 2020 to 28 February 2021; and period IV, from 1 March 2021 to 28 February 2022. The date 1 March 2020 was set as the cut-off date between prepandemic and pandemic years. Then, the two preceding and following calendar years were identified for the analysis in order to investigate four equally long time intervals. For each period, descriptive statistics were obtained using means and standard deviations for continuous variables and proportions for dichotomous and categorical variables. For the univariable analysis, the Kruskal–Wallis test was used to compare continuous variables across study periods, whereas Pearson’s chi-squared test was used for dichotomous and categorical variables. BW was categorized accord according to the Center for Disease Control/National Healthcare Safety Network (CDC/NHSN) classification [19]. As for exposure to antimicrobial agents, only those used as prophylaxis were considered (i.e., ampicillin, netilmicin, and fluconazole). Use of these antimicrobial agents was coded as (i) dichotomous (yes/no: yes was assigned in the case of having used the antimicrobial agent for at least one day) or (ii) cumulative (sum of the days of antimicrobial use). Similarly, use of devices (i.e., central line and mechanical ventilation) was coded as (i) dichotomous: (yes/no: yes was assigned in the case of having used a device for at least one day) or (ii) cumulative (sum of the days of device use). The in-hospital mortality rate and the HAI incidence rate together with their associated 95% confidence interval (CI) were calculated per 1000 patient days. Because multiple infection events were observed in a few patients, we also estimated the HAI infection rate per 1000 patient days accounting for recurrent events [20].

Time-to-first HAI (i.e., time-to-first CLABSI or VAP) was estimated by survival analysis. Firstly, we estimated the Kaplan–Meier survival function for each period of hospitalization, and survival curves were compared with the log-rank test. Then, the association between period of hospitalization and time-to-first event was assessed through a multivariable Cox regression model for proportional hazard, which provided estimates of the adjusted hazard ratio (aHR) and its associated 95% CI. The main exposure of interest, period of hospitalization, was adjusted for the potential confounders of the association based on expert knowledge [21]. As a result, the final model included the following variables: period of hospitalization (period II was set as the reference category, because it was the period immediately before the pandemic), sex (female vs. male), delivery (spontaneous vs. Cesarean section), birth weight in grams (because no infection was observed in higher BW classes, we used this variable as continuous), respiratory distress syndrome (yes/no), mechanical ventilation use in days (continuous, cumulative exposure in the time period from admission to discharge in patients without HAI or in the time period from admission to the day before HAI onset in patients with HAI), and previous use of netilmicin and fluconazole (yes/no: yes was assigned in the case of having used an antimicrobial agent for at least one day in the time period from admission to discharge in patients without HAI or in the time period from admission to the day before HAI onset in patients with HAI). Interaction terms between the variables were tested considering a *p*-value < 0.05 as cut-off. The proportionality assumption was checked by testing the statistical significance of interaction terms involving failure time, each one at a time. Multicollinearity was checked using as threshold a variance inflation factor of 5. All analyses were performed using STATA (StataCorp LLC, 4905 Lakeway Drive, College Station, TX, USA), version 17.0. A two-sided *p*-value < 0.05 was considered statistically significant.

## 3. Results

### 3.1. Characteristics of Patients

From 1 March 2018 to 28 February 2022, 564 neonates were hospitalized in the Umberto I teaching hospital of Rome, of which 503 were included in the HAI surveillance system (Table 1). The highest number of admissions occurred in period II with 148 patients, while period III had the lowest (N = 90). Total observation time ranged from 1629 days in period III to 2038 days in period I, with a longer average length of stay in the NICU in period III (18.1 days). A slightly higher proportion of male infants than females was found in periods III and IV, with 52 (57.8%) and 78 (59.1%) infants admitted, respectively, while the gestational age of the infants was similar across the four periods (about 33 weeks). Average BW was similar in periods I, II, and IV (1919.9 g, 2056.1 g, and 2025.7 g, respectively) and slightly lower in period III (1866.6 g). Considering BW classes, the most-represented category was 1501–2500 g, while the least-represented were 750 g or less and 751–1000 g. Regarding the delivery, most infants were born by Cesarean section, a proportion that reached 86.5% in period III. Preterm birth was quite common in all periods (around 80.0%). By contrast, respiratory distress syndrome increased over the years, ranging from 45.1% in period I to 62.9% in period IV, while twin pregnancy occurred less frequently (20–30% of cases).

As for the use of invasive devices, slightly more patients had a central line in periods I and II (69.9% and 64.9%, respectively) compared to periods III and IV (58.9% and 47.0%, respectively), but the average cumulative use in those who had a central line was highest in period III (19.6 days). Similarly, a lower number of patients underwent mechanical ventilation during the pandemic, especially in period IV (9.8%), but the highest average cumulative exposure was observed in period III (14.7 days). Antibacterial consumption was quite high; approximately three out of four patients were administered ampicillin, whereas two out of three were prescribed netilmicin in each period. However, while the cumulative average use of ampicillin was similar throughout the study, the average exposure to netilmicin was reduced in period IV. Fluconazole, on the other hand, was used in a lower proportion of patients (from 6.8% to 16.7%) with the highest average exposure in periods II and III. Lastly, a greater number of deaths were observed in periods I and II, accounting for slightly higher mortality rates (2.5 deaths per 1000 patient days (95% CI: 1.0–5.9) in period I and 3.5 per 1000 patient days (95% CI: 1.7–7.3) in period II).

### 3.2. Occurrence of HAIs

The prevalence of patients with at least one HAI was greater during the pandemic than prepandemic (3.0% in period I, 3.4% in period II, 11.1% in period III, and 6.8% in period IV), with the highest proportion of patients with at least one CLABSI or VAP in period III (Table 2). A total of four and five HAIs were recorded in periods I and II, respectively, whereas 16 and 11 HAIs were reported in periods III and IV, respectively. The most frequently diagnosed infection was CLABSI in all periods except March 2020–February 2021, in which 50% of infections were VAP. An increase in the incidence rate of HAIs was observed over time, with a peak in period III. After accounting for recurrent HAIs, similar rates were observed.

**Figure 1:**
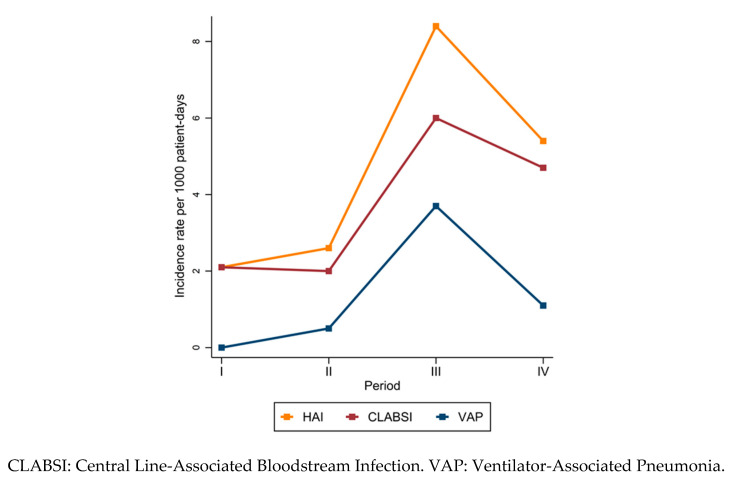
Incidence rate of first healthcare-associated infection (HAI) occurring in patients admitted to the neonatal intensive care unit of Umberto I teaching hospital of Rome between March 2018 and February 2022 by study period.

**Figure 2:**
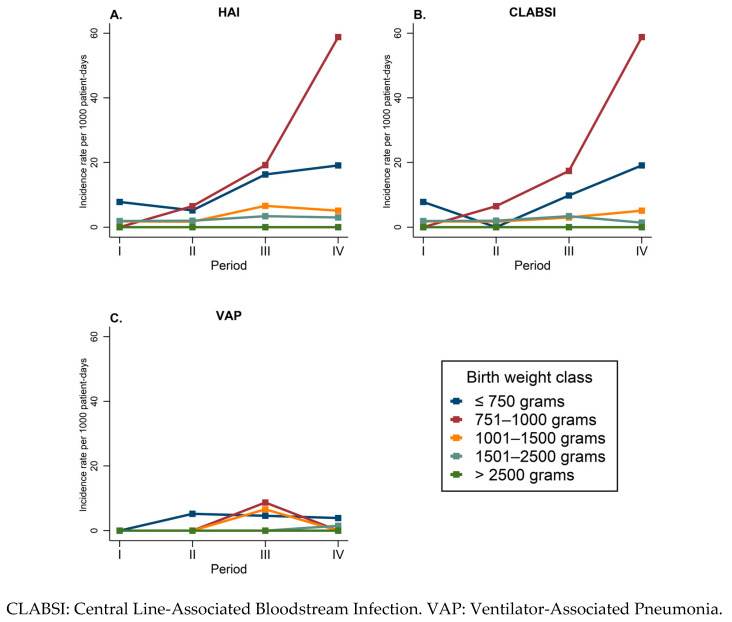
(**A**–**C**) Incidence rate of first healthcare-associated infection (HAI) occurring in patients admitted to the neonatal intensive care unit of Umberto I teaching hospital of Rome between March 2018 and February 2022 by study period.

Differentiating by type of HAI, the CLABSI and VAP incidence rates showed the same trend, with the highest values from March 2020 to February 2021 (Figure 1). However, when we stratified the HAI incidence rates by BW class, we found some differences: the lowest BW classes (≤750 g and 751–1000 g) had the highest rates of HAIs in period IV (Figure 2A), mostly CLABSI (Figure 2B); the middle BW classes (1001–1500 g and 1501–2500 g) showed a small peak in period III (Figure 2A), mainly VAP (Figure 2C) and CLABSI (Figure 2B), respectively. No HAI was diagnosed among patients in the highest BW class (>2500 g) in any period (Figure 2A–C).

**Figure 3:**
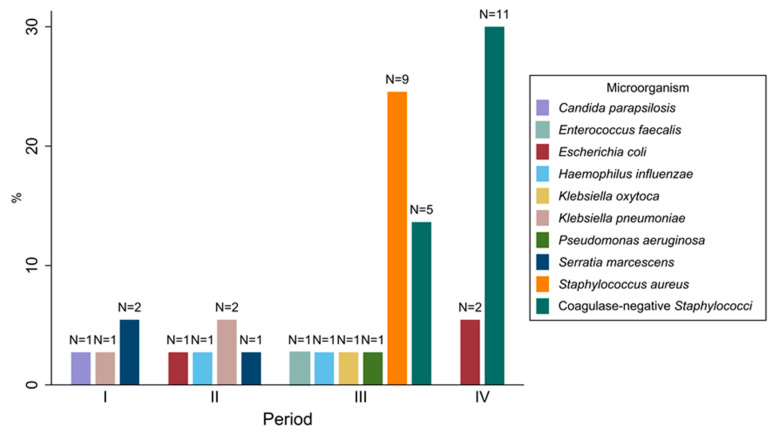
Frequency of isolation of microorganisms (N = 40) responsible for the healthcare-associated infections occurring in patients admitted to the neonatal intensive care unit of Umberto I teaching hospital of Rome between March 2018 and February 2022 by study period.

The etiology of HAIs across the four study periods varied (Figure 3). *Serratia marcescens* was the pathogen responsible for half the HAIs diagnosed in period I (5.0%), whereas *Klebsiella pneumoniae* was primarily detected in period II (5.0%). By contrast, *Staphylococcus aureus* (22.5%) and coagulase-negative *Staphylococci* (12.5%) were most frequently isolated in period III, as well as in period IV, where infections mainly involved coagulase-negative *Staphylococci* (27.5%), followed by *Escherichia coli* (5.0%). Other microorganisms, such as *Haemophilus influenzae*, were less frequently detected in periods II and III. As for microorganisms’ antimicrobial susceptibility patterns, out of 21 pathogens that could be classified according to the Magiorakos criteria [16], no multidrug resistant (MDR) microorganism was isolated in period I, whereas only one MDR isolate was found in period II (4.8%) and period IV (4.8%), respectively. Conversely, the greatest number of MDR microorganisms were isolated in period III (9 microorganisms, 42.9%), alongside an extensively drug-resistant (XDR) microorganism (4.8%). On the other hand, out of the 16 isolates of coagulase-negative *Staphylococci*, the number of such microorganisms resistant to oxacillin increased from 4 in period III (25.0%) to 10 in period IV (62.5%), while only 1 isolate was found to be resistant to both oxacillin and glycopeptides in period IV (6.3%).

### 3.3. Survival Analysis for First HAI

**Figure 4:**
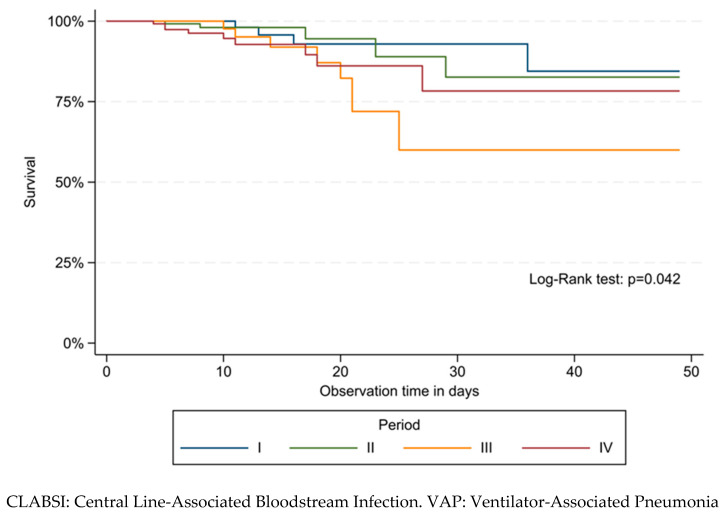
Kaplan–Meier survival estimates for time-to-first healthcare-associated infection (HAI) occurring in patients admitted to the neonatal intensive care unit of Umberto I teaching hospital of Rome between March 2018 and February 2022 by study period.

Kaplan–Meier estimates for the time of occurrence of the first HAI showed different survival curves across the study periods (*p* = 0.042) (Figure 4). Survival at 30 days decreased from 92.9% (95% CI: 79.3–97.7%) in period I to 82.6% (95% CI: 57.6–93.6%) in period II, and it was further reduced in periods III and IV (60.0%, 95% CI: 35.4–77.8% and 78.3%, 95% CI: 54.4–90.6%, respectively).

A multivariable analysis (Table 3) showed that being hospitalized during the pandemic periods was the main risk factor associated with the contraction of an HAI (period III, aHR: 4.88, 95% CI: 1.33–17.97; period IV, aHR: 6.45, 95% CI: 1.53–27.24). Also, mechanical ventilation (aHR: 1.04, 95% CI: 1.02–1.06) was positively associated with the outcome. By contrast, having a higher BW seemed to be a protective factor against HAIs (aHR: 0.99, 95% CI: 0.98–0.99). Sex, type of delivery, respiratory distress syndrome, and previous use of netilmicin and/or fluconazole did not appear to influence HAI onset.

## 4. Discussion

Among the indirect consequences of the COVID-19 pandemic, an increase in the incidence of HAIs has been frequently reported, especially in some wards, such as ICUs [11,22]. However, most studies have focused on adult ICUs, while data on the impact of the pandemic on HAIs in NICUs are still scarce. This is probably because neonates have been less affected by the SARS-CoV-2 infection, and the reorganization of NICUs was less pronounced than in adult ICUs, which needed to cope with a high number of critically ill patients [23]. Among others, starting from March 2020, our NICU limited parent visits as much as possible to avoid overcrowding and reduce the risk of contagion for infants and healthcare personnel. At the same time, the use of personal protective equipment during patient management was mandatorily implemented. In addition, a surveillance protocol based on periodical nasopharyngeal swabs for SARS-CoV-2 detection was established for both parents and healthcare staff. However, in line with the results of Kharrart et al. [8], we recorded an increase in the HAI incidence rate in our NICU during the pandemic, with a peak in the first year (March 2020–February 2021). Therefore, concurrently with the measures taken to control the spread of the virus during the COVID-19 pandemic, it appears that some factors led to less attention being paid to procedures designed to prevent and control traditional HAIs, negatively affecting their incidence. Furthermore, the deficit in individual preventive devices, particularly during the early months of the pandemic, may have increased the fear of infection transmission thereby influencing the implementation of prevention and control measures [24]. Alternatively, or in addition, the increased demand for staff needed to manage COVID-19 patients led hospitals to reorganize their facilities to meet clinical needs [25], and these adaptations may have forced hospitals to hire new healthcare staff, including inexperienced personnel. When this happens in departments such as the NICUs, the shortage of experienced and qualified staff can result in adverse outcomes on newborns, including higher rates of HAIs, as previously documented [26,27]. However, further investigation is needed to determine the specific impact that the discussed factors may have had on HAI acquisition in NICU settings.

As for the HAI type, both CLABSI and VAP rates increased during the pandemic, even though the CLABSI increment was not significant in the univariate analysis, probably due to reduced statistical power. Indeed, despite the lower number of neonates using central lines in these years, the higher average exposure, especially in period III, may at least partially explain the peak in CLABSI incidence recorded between March 2020 and February 2021 [4,28,29]. In addition, period III witnessed a *S. aureus* outbreak in our NICU that accounted for most VAP recorded between May and July 2020. Gram-negative bacteria, particularly *S. marcescens* and *K. pneumoniae*, were the pathogens most frequently responsible for HAIs before the pandemic, in line with data from the literature in which they were often involved in NICU outbreaks [30,31]. In contrast, during the pandemic years, the most frequently detected pathogens were MDR *S. aureus* and oxacillin-resistant coagulase-negative *Staphylococci*, usually the main causes of HAIs in infants [29,32,33]. This change in the microorganism breakdown may help explain why, although more HAIs occurred in periods III and IV, the infant mortality rate did not increase over this time. In fact, the Gram-negative bacteria that circulated in the first two years of surveillance are known to be potentially fatal in NICUs [34,35,36,37] compared to the Gram-positive bacteria found in the other two periods, whose infections have recently become more manageable and are less likely to result in patient death [38].

However, our study confirmed that a major risk factor for the occurrence of HAIs in NICUs was low BW [39,40]. This is because such infants are particularly vulnerable to bacterial infections given their immature immune system development, need for prolonged hospitalizations, and need for monitoring, testing, and invasive treatments that circumvent the skin barrier’s defense mechanisms [41,42,43,44]. This applies to both the lowest BW classes, that accounted for most infections registered throughout the study period, with the highest HAI incidence rates recorded in the 751–1000 g class probably being the result of a smaller number of patients days spent under surveillance compared to the ≤750 g class. Furthermore, while our results show that prolonged mechanical ventilation, which requires the use of breathing circuits known to be important sources and breeding grounds of pathogenic microorganisms [45], contributed to the occurrence of HAIs, variables related to the patients’ demographic characteristics and clinical conditions did not influence the outcome. These results partially contrast with those studies in which the male sex and Cesarean section were found to increase HAI occurrence [46,47] but align with findings in which respiratory distress did not show a direct association with HAI onset [47,48]. As for antimicrobials, even though their use did not seem to affect HAI acquisition in our sample, it is worth mentioning that they should be carefully prescribed, because their continued consumption, when inappropriate, can lead to adverse events, including the selection and emergence of highly resistant bacteria [49].

This study has several strengths and limitations. The main strength is the ability to compare data over time. Because data were collected as part of a four-year continuous surveillance system routinely carried out by the Department of Public Health and Infectious Diseases, a potential bias in the results due to overworked NICU staff is unlikely. In addition, to the best of our knowledge, this is one of the few studies that describes and identifies risk factors related to HAI occurrence in a NICU during an emergency period, such as the COVID-19 pandemic. In this regard, we plan to conduct new studies in the near future on the occurrence of HAIs in NICU and ICU settings as the pandemic progresses. In contrast, the first limitation is represented by the fact that we do not have data on the SARS-CoV-2 positivity or negativity status of the infant mothers, even though all hospitalized infants were tested and were SARS-CoV-2 negative. Second, patients discharged from the NICU were no longer under surveillance, although only the most stable patients were chosen for transfer. Third, even though we adjusted for the main risk factors, namely demographic characteristics and use of invasive device and antibiotics, we may have not fully accounted for clinical severity, meaning that some residual confounders may be still present. However, this bias is likely to be constant across time periods. Lastly, we did not study the impact of HAIs on patient mortality, although this was not a goal of our research. Further studies should be conducted to address this issue, together with research that analyzes hand hygiene compliance, which could be of interest to better understand the mechanisms behind any increase or decrease in the incidence rates.

## 5. Conclusions

We found higher rates of HAIs in our NICU during the COVID-19 pandemic. This, coupled with the fact that the microorganisms involved were different across the study period, suggests a crucial role for patient management and underlines the importance of implementing effective HAI prevention and control strategies [50,51]. Because it is widely recognized that hand hygiene is a highly effective tool in the prevention and control of HAIs [52], it is recommended that further efforts be made to promote adherence to hygiene precautions and increase knowledge and awareness of these issues among NICU healthcare workers.

## Figures and Tables

**Table 1:** Key characteristics of the patients admitted to the neonatal intensive care unit of Umberto I teaching hospital of Rome between March 2018 and February 2022 (N = 503) by study period. Results are expressed as number (percentage, %) or mean (standard deviation, SD).

	Period				
	I	II	III	IV	
	1 March 2018 to 28 February 2019	1 March 2019 to 29 February 2020	1 March 2020 to 28 February 2021	1 March 2021 to 28 February 2022	*p*-Value *
Patients, N	133	148	90	132	
Total observation time, patient days	2038	1999	1629	1925	
Length of NICU stay in days, mean (SD)	15.3 (15.8)	13.5 (15.6)	18.1 (21.7)	14.6 (15.6)	0.470
Sex, N (%)					0.440
Female	61 (45.9)	74 (50.0)	38 (42.2)	54 (40.9)	
Male	72 (54.1)	74 (50.0)	52 (57.8)	78 (59.1)	
Gestational age in weeks, mean (SD) (N = 499)	32.9 (4.3)	33.5 (4.0)	32.8 (4.2)	33.6 (3.7)	0.300
Birth weight in grams, mean (SD)	1919.9 (885.4)	2056.1 (871.6)	1866.6 (881.1)	2025.7 (764.1)	0.180
Birth weight class, N (%)					0.450
≤750 g	7 (5.3)	6 (4.1)	9 (10.0)	8 (6.1)	
751–1000 g	8 (6.0)	8 (5.4)	6 (6.7)	1 (0.8)	
1001–1500 g	32 (24.1)	29 (19.6)	19 (21.1)	27 (20.5)	
1501–2500 g	56 (42.1)	63 (42.6)	36 (40.0)	61 (46.2)	
>2500 g	30 (22.6)	42 (28.4)	20 (22.2)	35 (26.5)	
Delivery, N (%)					0.810
Spontaneous	23 (17.4)	26 (17.8)	12 (13.5)	20 (15.4)	
Cesarean section	109 (82.6)	120 (82.2)	77 (86.5)	110 (84.6)	
Preterm birth, N (%)	105 (78.9)	114 (77.0)	74 (82.2)	106 (80.3)	0.800
Twin pregnancy, N (%)	22 (16.5)	26 (17.6)	25 (27.8)	33 (25.0)	0.094
Respiratory distress syndrome, N (%)	60 (45.1)	79 (53.4)	55 (61.1)	83 (62.9)	0.018
Use of central line, N (%)	93 (69.9)	96 (64.9)	53 (58.9)	62 (47.0)	<0.001
Cumulative days of central line, mean (SD) (N = 304)	11.9 (10.1)	13.3 (16.5)	19.6 (21.4)	13.9 (14.8)	0.350
Use of mechanical ventilation, N (%)	34 (25.6)	38 (25.7)	21 (23.3)	13 (9.8)	0.003
Cumulative days of mechanical ventilation, mean (SD) (N = 106)	7.6 (8.5)	8.6 (15.3)	14.7 (20.4)	10.8 (11.7)	0.370
Use of ampicillin, N (%)	107 (80.5)	116 (78.4)	67 (74.4)	97 (73.5)	0.510
Cumulative days of ampicillin use, mean (SD) (N = 387)	6.9 (2.8)	6.8 (3.1)	6.4 (2.7)	6.0 (2.7)	0.093
Use of netilmicin, N (%)	102 (76.7)	102 (68.9)	61 (67.8)	94 (71.2)	0.420
Cumulative days of netilmicin use, mean (SD) (N = 359)	5.3 (2.2)	5.5 (2.5)	5.8 (2.5)	4.6 (2.3)	<0.001
Use of fluconazole, N (%)	18 (13.5)	14 (9.5)	15 (16.7)	9 (6.8)	0.091
Cumulative days of fluconazole use, mean (SD) (N = 56)	17.3 (12.0)	30.1 (19.5)	34.2 (19.1)	22.2 (16.4)	0.043
NICU deaths, N (%)	5 (3.8)	7 (4.7)	2 (2.2)	4 (3.0)	0.760
NICU mortality, rate × 1000 patient days (95% CI)	2.5 (1.0–5.9)	3.5 (1.7–7.3)	1.2 (0.3–4.9)	2.1 (0.8–5.5)	

NICU: Neonatal Intensive Care Unit. CI: Confidence Interval. HAI: Healthcare-Associated Infection. * Pearson’s chi-squared test was used for categorical variables and Kruskal–Wallis test for continuous variables.

**Table 2:** Key characteristics of healthcare-associated infections (HAIs) diagnosed (N = 36) among patients admitted to the neonatal intensive care unit of Umberto I teaching hospital of Rome between March 2018 and February 2022 (N = 503) by study period. Results are expressed as number (percentage, %) or mean (standard deviation, SD).

	Period				
	I	II	III	IV	
	1 March 2018 to 28 February 2019	1 March 2019 to 29 February 2020	1 March 2020 to 28 February 2021	1 March 2021 to 28 February 2022	*p*-Value *
Patients, N	133	148	90	132	
Patients with at least one HAI, N (%)	4 (3.0)	5 (3.4)	10 (11.1)	9 (6.8)	0.034
Patients with at least one CLABSI, N (%)	4 (3.0)	4 (2.7)	8 (8.9)	8 (6.1)	0.100
Patients with at least one VAP, N (%)	0 (0.0)	1 (0.7)	5 (5.6)	2 (1.5)	0.007
HAIs, N	4	5	16	11	
Type of HAI, N (%)					0.130
CLABSI	4 (100.0)	4 (80.0)	8 (50.0)	9 (81.8)	
VAP	0 (0.0)	1 (20.0)	8 (50.0)	2 (18.2)	
Incidence rate for first HAI × 1000 patient days (95% CI)	2.1 (0.8–5.5)	2.6 (1.1–6.3)	8.4 (4.5–15.6)	5.4 (2.8–10.4)	
Incidence rate for recurrent HAIs × 1000 patient days (95% CI)	2.0 (0.7–5.2)	2.5 (1.0–6.0)	9.4 (5.5–16.0)	5.6 (3.1–10.3)	

CI: Confidence Interval. CLABSI: Central Line-Associated Bloodstream Infection. VAP: Ventilator-Associated Pneumonia. * Pearson’s chi-squared test.

**Table 3:** Multivariable Cox regression model for first healthcare-associated infection among patients admitted to the neonatal intensive care unit of Umberto I teaching hospital of Rome between March 2018 and February 2022 (N = 503).

	aHR	95% CI	*p*-Value
Period			
II (1 March 2019 to 29 February 2020)	Ref.		
I (1 March 2018 to 28 February 2019)	1.62	0.34–7.67	0.544
III (1 March 2020 to 28 February 2021)	4.88	1.33–17.97	0.017
IV (1 March 2021 to 28 February 2022)	6.45	1.53–27.24	0.011
Sex			
Female	Ref.		
Male	1.08	0.49–2.37	0.843
Delivery			
Spontaneous	Ref.		
Cesarean section	0.73	0.27–1.96	0.537
Birth weight, grams	0.99	0.98–0.99	0.030
Respiratory distress syndrome			
No	Ref.		
Yes	1.86	0.76–4.52	0.172
Mechanical ventilation use, days	1.04	1.02–1.06	<0.001
Previous use of netilmicin			
No	Ref.		
Yes	5.23	0.65–42.40	0.121
Previous use of fluconazole			
No	Ref.		
Yes	0.89	0.33–2.44	0.828

aHR: adjusted Hazard Ratio. CI: Confidence Interval.

## Data Availability

The data sets generated and analyzed during the current study are available from the corresponding author on reasonable request.
